# Arbroath Infirmary

**Published:** 1898-09-10

**Authors:** 


### ARBROATH INFIRMARY.

### HOSPITAL DISPENSERS AND THE PHARMACY ACT.

A dispute has arisen at Arbroath Infirmary with reference to the dispensing, which has for many years been in the capable hands of Miss Kay. The local pharmacists are at length up in arms about the matter, and a correspondence has taken place between their association and the directors of the infirmary. The substance of the chemists' complaint is that Miss Kay "possesses no such qualifications as are by statute required of practising dispensing chemists," to which that lady replies that she never "professed to be a practising dispensing chemist." Is there then any such statutory qualification? It is true that Poor Law dispensers must possess one or other of the qualifications prescribed by the Local Government Board, which would, no doubt, exclude persons in Miss Kay's position. But they are, in effect, Government officials. In private and semi-public infirmaries, many of which are charities voluntarily supported, it does not appear that such qualification is essential, and unless the dispensers call themselves chemists, or use any of the titles enumerated in the Pharmacy Act, 1868, there seems to be nothing to prevent them dispensing drugs without being legally qualified. So far, Miss Kay's position is a strong one. The next point is, however, a more difficult one to dispose of. The chemists say that at this infirmary scheduled poisons are dispensed both to in-patients and out-patients by this lady, who, it is admitted, is not a person legally entitled to sell such poisons. But does she sell them? Or does she "keep open shop for selling or dispensing" them? Such drugs, whether poisonous or not, as are not charged to the patients are not sold. On the other hand, we hardly think a dispenser at an infirmary can be said to "keep an open shop for dispensing poisons." The phrase imports the notion of sale, though it may be anomalous to prohibit sales and allow free distribution. However, the Pharmacy Act is a penal one, and must be construed strictly. It is not "dispensing," but "keeping open shop for dispensing" poisons that is prohibited by that Act. Therefore, although the medicines are dispensed, unless the dispensary is open to anyone as a shop for the sale of poisons, which does not appear to be the case here, we do not think the dispenser can be said to "keep open shop" for their sale. She is merely a compounder of the drugs ordered by the infirmary doctor for the in and out patients. Miss Kay rather suggests that she is protected in dispensing poisons on a medical prescription. If she refers to the exemption in sec. 17 of the Pharmacy Act, this has no application to the case; first, because that section only relates to labelling, &c.; and, secondly, the dispensing must be by a person registered under that Act. She is, however, no doubt right in asking the question whether "selling" means "distributing." We have already indicated our opinion that it does not. It would, in fact, be entirely contrary to the grammatical meaning of the words to say that "selling" and "giving" are synonymous terms. Even where a nominal inclusive charge is made for advice and medicine we do not think the dispenser can be said to "sell" the medicines, as there is no appropriation of any part of the charge to the price of the drugs. So far, then, as we interpret the Pharmacy Act, we do not think that Miss Kay has brought herself within any statutory prohibition. As a general rule, it must be conceded, dispensers at infirmaries should hold a recognised qualification; but Miss Kay's case is quite exceptional, and inasmuch as she has for many years discharged her duties with perfect satisfaction, both to her employers and patients, we should be sorry to hear of her supersession on purely formal grounds.

